# An Unusual Case of Solitary Osteochondroma of the Iliac Wing

**DOI:** 10.1155/2020/8831806

**Published:** 2020-12-18

**Authors:** Christopher Thomas, Brent Sanderson, Dennis G. Horvath, Michael Mouselli, Janet Hobbs

**Affiliations:** ^1^Community Memorial Health System-Department of Orthopaedic Surgery, 147 N. Brent St. Ventura, CA 93003, USA; ^2^Graduate Medical Education, Community Memorial Health System 147 N Brent St. Ventura, CA 93003, USA; ^3^GME Department, Community Memorial Hospital, 147 N. Brent St. Ventura, CA 93003, USA

## Abstract

**Introduction:**

Osteochondromas represent one of the most common bone tumors accounting for 8% of all bone tumors. While most osteochondromas arise in the metaphysis of long bones, osteochondromas have been reported in atypical locations such as the scapula, metatarsals, and the pelvic region. Osteochondromas are capable of growing large enough to cause mass effects and can undergo malignant transformation, stressing the clinical importance of recognizing these tumors. *Case Presentation*. In this case, we present an 18-year-old skeletally mature Caucasian male with a symptomatic osteochondroma arising from the iliac wing. The osteochondroma increased in size since he reached skeletal maturity. This resulted in a mass effect that interfered with activities of daily living, including clothing wear and symptomatic impaction on hard surfaces.

**Conclusion:**

The majority of osteochondromas arise from the metaphysis of long bones, but case reports have shown that osteochondromas presenting in atypical locations such as the pelvis do occur. In the case of our patient, his asymptomatic pelvic tumor grew to the extent that it was causing interference with activities of daily living. Surgical excision of his tumor proved to be curative, and there was no recurrence at 6 months after excision. Osteochondromas in this region are capable of growing large enough to cause sexual dysfunction. Clinical suspicion must be high to properly diagnose osteochondromas in atypical locations. All providers, particularly those in primary care, should be aware of these locations as patients with symptomatic mass lesions will likely initially present here.

## 1. Introduction

Osteochondromas represent one of the most common bone tumors accounting for 8% of all bone tumors [1]. Although common, the reported incidence may be underestimated due to the often clinically silent presentation of this bone tumor [1]. These tumors are comprised of subperiosteal bone projections which are superficially covered by a cartilaginous cap [1].

Osteochondromas most commonly arise as nonhereditary solitary lesions. However, about 15% of osteochondromas arise from an autosomal dominant disorder known as Hereditary Multiple Osteochondromas (H.M.O.) [2]. Heparin sulfate is important for skeletal processes such as skeletogenesis and skeletal growth [3]. Mutations in the tumor suppressor genes EXT1 and EXT2 lead to heparan sulfate deficiencies causing HMO [3].

Osteochondromas typically form in bones which undergo endochondral ossification with the most common sites affected being the metaphysis of the distal femur, proximal humerus, and proximal tibia and fibula [1]. However, osteochondromas have been reported in atypical locations such as the scapula, metatarsals, and the pelvic region [4–6]. Osteochondromas are typically benign and have been known to undergo spontaneous shrinkage [7], although the benign nature is not always the case as osteochondromas can undergo a malignant transformation in up to 1% of solitary lesions and up to 3% in patients with multiple lesions [1]. When left alone, osteochondromas can grow large enough to impinge on important vessels leading to vascular complications [8, 9]. These complications demonstrate the importance of recognizing osteochondromas in clinically silent patients and those with atypical locations. In this case, we present an 18-year-old skeletally mature individual with a symptomatic osteochondroma arising from the iliac wing.

## 2. Case Presentation

An 18-year-old male presented to the outpatient orthopedic surgery clinic for evaluation of a painful left iliac wing mass. The mass was first identified by the patient three years prior and had an insidious onset that continued to increase in size. The patient's primary care physician ordered an X-ray of the pelvis to evaluate the patient's left iliac mass.

The patient denied any preceding unintentional weight loss, whole body aches, fatigue, and night sweats over that time span. Past medical history is complicated by insomnia secondary to his history of depression and anxiety. There was no history of trauma to the area. The patient reported the mass as nontender to direct palpation but complained he occasionally hit the mass against objects during everyday activity which results in subjective pain and tenderness. He also complained that the mass catches on his clothing and makes wearing a belt difficult. He denies any neurologic changes or deficit. He has no family history of bone tumor, M.H.E., and osteochondromas.

On examination, there was a nontender hard mass and area of swelling (approximately 4 cm by 3 cm measure by ruler) located posterosuperior to the left anterior superior iliac spine (ASIS) of the iliac wing. Direct palpation of the mass demonstrated that it was fixed to the underlying bone while the skin was free and mobile over the mass. The patient did not note any other masses or areas of concern on the exam.

**Figure 1:**
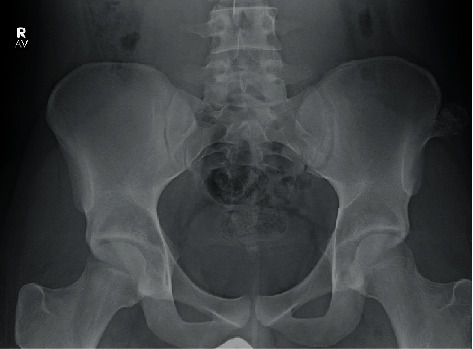
Preoperative anterosuperior pelvis plain-film radiograph. Radiograph demonstrates an exophytic mass growing laterally from the left iliac wing measuring 2.8 × 3.1 cm (Osteochondroma pre-op.jpg).

Plain X-rays of the A.P. pelvis demonstrated a 2.8 × 3.1 cm bony growth exostosis along the lateral aspect of the left iliac wing consistent with an osteochondroma (Figure 1).

**Figure 2:**
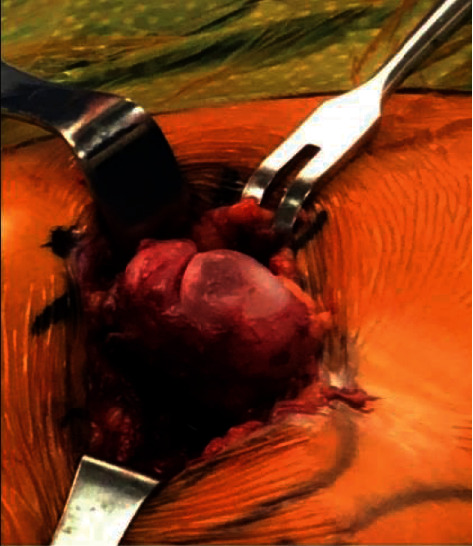
Preoperative coronal T1 M.R.I. M.R.I. demonstrates osseous exostosis with a 5 mm cartilaginous cap growing laterally from the left iliac wing. The exostosis shows medullary continuity with underlying bone consistent with osteochondroma (Osteochondroma pre-op Coronal T1 MRI.jpg).

M.R.I. was completed and demonstrated a mass arising from the lateral aspect of the left iliac wing, 2.6 × 2.4 cm in dimension. The osseous exostosis shows cortical and medullary continuity with the underlying bone. There is a 5 mm cartilaginous cap as well as mild edema just beneath the cartilage cap (Figure 2). There is a mild enhancement of the distal aspect of the exostosis as well as within the surrounding soft tissues. Mild edema was present within adjacent soft tissues.

## 3. Surgical Technique

**Figure 3:**
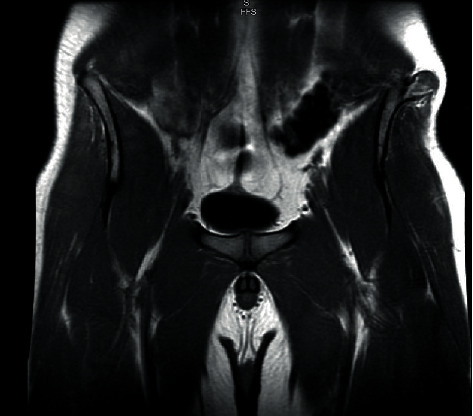
Intraoperative photograph of anterior left pelvis. The photograph demonstrates a pedunculated exophytic mass growing from the left iliac wing. The mass is superficially covered by a cartilaginous cap which can be appreciated surrounding the cortical bone (Osteochondroma Intra operative Left anterior hip.jpg).

A 7 cm incision was made directly over the mass at the anterior aspect of the left iliac crest. Sharp dissection was performed through the subcutaneous tissue. Electrocautery was used for hemostasis. Blunt dissection was used to establish fascial planes. The encapsulated mass was then encountered. The capsule was opened, which demonstrated the cartilaginous cap (Figure 3).

The muscular tissue was then elevated off the stalk with electrocautery attempting to preserve as much native tissue as possible. The periosteum was elevated around the base of the mass to fully appreciate the size and dimensions. Meticulous hemostasis was maintained throughout the case. Once adequate visualization of the mass was achieved, a curved osteotome was used to sharply transect the mass at the base of the pedunculate stalk in line and flush with the contour of the iliac wing. The specimen was sent immediately to pathology for permanent sections. The site was then inspected for any remaining mass and to ensure it was completely removed en bloc. Bone wax was then placed over the cancellous bone to help prevent hematoma formation and provide adequate hemostasis. Layered closure was subsequently completed.

## 4. Pathology

**Figure 4:**
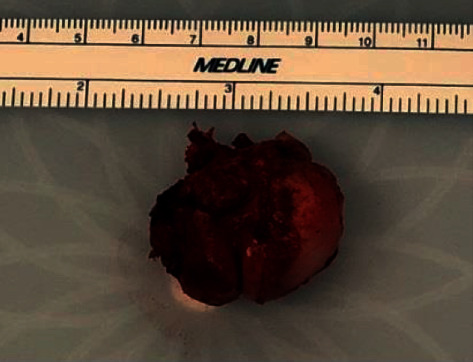
Intraoperative osteochondroma gross specimen. This photograph demonstrates the solitary lesion after it was fully excised from the pelvis (Osteochondroma Gross Specimen.jpg).

The lobulated firm mass removed was measured to be 3.0 × 2.0 × 1.6 cm. The resection margin was smooth and is inked blue. The capsule is inked black (Figure 4).

The specimen is sectioned to reveal a tan-white smooth firm cut surface. Histopathology confirmed the mass to be osteochondroma without any undifferentiated cells. No malignancy was identified.

## 5. Postoperative Follow-Up

**Figure 5:**
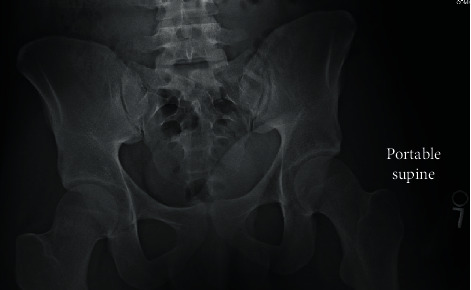
6-month postoperative anteroposterior X-ray of the pelvis. This radiograph demonstrates complete excision of the exophytic mass from the patient's left iliac wing without any sign of recurrence (Osteochondroma 6 month post-op follow up.jpg).

The patient presented 6 months postoperatively for a follow-up visit. The patient reported no pain and a well-healed scar, and there was no recurrence of growth on X-ray (Figure 5).

## 6. Discussion

Osteochondromas represent one of the most common benign bone lesions occurring in about 3% of the population and causing complications such as vascular compromise, neurologic defects, and compartment syndrome in up to 4% of cases [10]. When multiple lesions are present, osteochondromas exist as part of a syndrome known as H.M.O. which is related to mutations in EXT1 and EXT2 genes [3]. However, solitary lesions are the most common presentations accounting for 85% of all osteochondromas [1]. The majority of osteochondromas arise from the metaphysis of long bones, but case reports have shown that osteochondromas presenting in atypical locations do occur [4, 6, 11].

Radiology is typically diagnostic of osteochondromas, with characteristic features that can be seen on multiple imaging modalities. Osteochondromas typically show continuity with underlying bone marrow and cortices [1]. This was the case with our patient, as evidenced on M.R.I. (Figure 2). These lesions are covered by a cartilaginous cap, which is usually thin compared to the osseous component. When the cartilaginous cap is thickened to greater than 20 mm, malignant transformation is suspected [1]. Our patient presented with a symmetric cartilaginous cap of 5 mm on M.R.I. (Figure 2). Pathology reports confirmed there was no malignant transformation after the mass was completely excised, consistent with what is expected from cartilage cap size.

Surgical excision of these lesions is typically curative, and recurrence of osteochondromas can indicate either incomplete removal or malignant transformation [1]. In the case of solitary lesions, approximately 1% of all osteochondromas undergo malignant transformation [1]. While surgery is typically curative, nonsurgical options have also shown to be effective. In a report of 17 patients in Japan that presented with osteochondromas, 8 of the cases underwent spontaneous shrinkage [7]. Osteochondromas with sessile morphology were more likely to undergo shrinkage as compared to pedunculated morphology [7]. As all surgeries have well-described risks, this raises the question of what is the best treatment for these masses. In the case of our patient, the pedunculated morphology of the mass meant it was less likely to undergo spontaneous shrinkage. In our case study, the tumor size was increasing, which began causing significant impairment with daily living. We felt that surgical excision was the appropriate management of our young healthy patient's enlarging osteochondroma.

Our 18-year-old male presented with a painless lesion on his left iliac wing, which was growing in size for the past 3 years. This asymptomatic presentation is the typical way in which osteochondromas present [1]. The lack of symptoms allows for osteochondromas to grow until they become noticed from their mass effect. When these secondary effects become noticed, they can present in a multitude of ways. These include complications such as dysphagia, Kienbock's disease, and hemothorax [12–14]. In the case of our patient, his initially asymptomatic pelvic tumor grew to the extent that it was causing interference with activities of daily living. Osteochondromas in this region are capable of growing large enough to cause sexual dysfunction [15]. These symptoms highlight the clinical importance to all practitioners, particularly those in a primary care setting where a patient with these complaints may initially present.

## 7. Conclusion

The majority of osteochondromas arise from the metaphysis of long bones, but case reports have shown that osteochondromas presenting in atypical locations such as the pelvis do occur [4, 6, 11]. In the case of our patient, this asymptomatic pelvic tumor grew to the extent that it was causing interference with activities of daily living. Surgical excision of his tumor proved to be curative, and there was no recurrence at 6 months after excision. Osteochondromas in this region are capable of growing large enough to cause sexual dysfunction [15]. Clinical suspicion must be high to properly diagnose osteochondromas in atypical locations. All providers, particularly those in primary care, should be aware of these locations as patients with symptomatic mass lesions will likely initially present here.
